# CCRaVAT and QuTie - enabling analysis of rare variants in large-scale case control and quantitative trait association studies

**DOI:** 10.1186/1471-2105-11-527

**Published:** 2010-10-21

**Authors:** Robert Lawrence, Aaron G Day-Williams, Katherine S Elliott, Andrew P Morris, Eleftheria Zeggini

**Affiliations:** 1 Wellcome Trust Centre for Human Genetics, University of Oxford, Oxford, UK; 2 Wellcome Trust Sanger Institute, Hinxton, UK

## Abstract

**Background:**

Genome-wide association studies have been successful in finding common variants influencing common traits. However, these associations only account for a fraction of trait heritability. There has been a shift in the field towards studying low frequency and rare variants, which are now widely recognised as putative complex trait determinants. Despite this increasing focus on examining the role of low frequency and rare variants in complex disease susceptibility, there is a lack of user-friendly analytical packages implementing powerful association tests for the analysis of rare variants.

**Results:**

We have developed two software tools, CCRaVAT (Case-Control Rare Variant Analysis Tool) and QuTie (Quantitative Trait), which enable efficient large-scale analysis of low frequency and rare variants. Both programs implement a collapsing method examining the accumulation of low frequency and rare variants across a locus of interest that has more power than single variant analysis. CCRaVAT carries out case-control analyses whereas QuTie has been developed for continuous trait analysis.

**Conclusions:**

CCRaVAT and QuTie are easy to use software tools that allow users to perform genome-wide association analysis on low frequency and rare variants for both binary and quantitative traits. The software is freely available and provides the genetics community with a resource to perform association analysis on rarer genetic variants.

## Background

Recent advances in high-throughput genotyping have made large-scale genetic association studies possible. Genome-wide association studies (GWAS) for complex disease have met with unprecedented success in identifying common susceptibility variants. However, the discovered common-frequency single nucleotide polymorphism (SNP) associations do not account for a large proportion of the genetic component of disease. The field is now focusing on the analysis of low frequency and rare variants (i.e. minor allele frequency (MAF) ≤0.05) to investigate if they will help explain the missing heritability in complex trait etiology [1,2]. While the sample sizes currently investigated are large enough for a well-powered GWAS of common variants, they are not large enough to provide sufficient power for the single-point analysis of low frequency/rare variants with small to moderate effect sizes [3]. We have developed association analysis software, CCRaVAT (Case-Control Rare Variant Analysis Tool) and QuTie (Quantitative Trait), which allow the large-scale analysis of low frequency/rare polymorphisms. The software increases power over single marker analysis of these variants by pooling the low frequency/rare variants within defined regions and treating them as a single "super-locus" [3,4]. These software tools are suitable for the analysis of SNP data from both commercial GWAS platforms as well as of variants discovered from resequencing projects. The programs find loci where the low frequency/rare variant content is significantly different between cases and controls, or where the means of a quantitative trait differ between groups with and without these variants.

## Implementation

CCRaVAT and QuTie are Linux command-line based utilities written in Perl. The scripts utilize the GetOpt, POSIX, and GD Perl modules. The GD module is necessary to produce the graphical output, and the POSIX module is used to calculate the logarithm base 10 of the p values. The tools have been tested on a variety of GWAS datasets and the system requirements depend mainly on the size of the study (i.e. number of SNPs and individuals genotyped). The software requires that the data be separated by chromosome for efficiency. For a genome-wide dataset separated by chromosome consisting of 450,000 SNPs typed in 5,000 individuals, CCRaVAT requires ~200 Mb of RAM. The software development and testing of the applications were performed on machines with dual-core Athlon processors. The scripts can take a variable amount of time to run depending on the options used. The run time for a typical gene-centric genome-wide analysis, using approximately 450,000 SNPs and 5,000 individuals separated by chromosome, is less than 24 hours. Permutation testing can add considerably to the computing time depending on the number of regions analyzed and the numbers of permutations run.

## Results and Discussion

The statistical properties of the low frequency/rare variant collapsing (super-locus) association test that we have implemented have been described previously [3,4]. Although methods for how to analyze low frequency/rare variants have been developed, to our knowledge there are no published software packages that implement them. This lack of software tools motivated the development of CCRaVAT and QuTie.

### Analytical Framework

**Figure 1:**
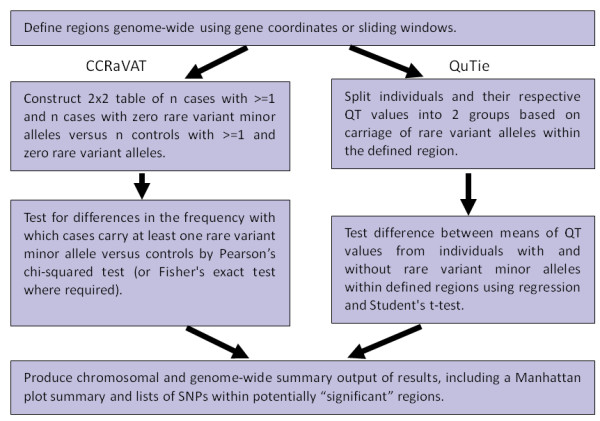
**CCRaVAT and QuTie Workflow**. Flowchart summarizing the implementation of the low frequency/rare variant analysis methods in CCRaVAT and QuTie.

Figure 1 provides an overview of the analytical approach implemented in CCRaVAT and QuTie. The first step in implementing the collapsing approach involves the definition of regions in which low frequency/rare variants are collapsed. These chromosomal regions can either be defined by sliding windows of predefined length across the genome or genic regions defined by intervals either side of the transcriptional start and stop sites of genes. CCRaVAT and QuTie differ in the study designs analyzed and statistical techniques used to determine the significance of the comparison. CCRaVAT analyzes binary trait data and constructs a 2 x 2 contingency table of the presence or absence of low frequency/rare variant minor alleles in cases and controls for each region. Differences in the proportion of cases and controls carrying low frequency/rare variant minor alleles are tested using a Pearson's chi-squared test or a Fisher's exact test. CCRaVAT also allows users to generate empirical p values by permuting case-control status a predefined number of times and repeating the analysis for each replicate. QuTie implements the analysis of quantitative traits in a sample of unrelated individuals and analyzes the differences in quantitative trait means for individuals carrying at least one low frequency/rare variant minor allele and individuals carrying no low frequency/rare variant minor alleles within the defined region. The quantitative trait values in the two groups are compared using linear regression and a Student's t-test. The analysis methods assume all individuals are unrelated.

### Input Files

CCRaVAT and QuTie require two input files per chromosome: a map file and a pedigree file. The map file contains information about the markers analyzed and their position along the chromosome. CCRaVAT and QuTie allow both a 3 column and a 4 column formatted map file, as seen in Table 1. The 3 column map file illustrated in Table 1A contains information on chromosome, marker name, and base pair (bp) position of analyzed markers. The 4 column map file shown in Table 1B is the map file format used by the program PLINK [5] and contains the chromosome, marker name, genetic position and bp position of analyzed markers. The pedigree file holds information about the individuals and their genotypes. The pedigree file is a white-space delimited (space or tab) file that needs to be in the standard pre-Makeped linkage format described and illustrated in Table 2. If performing a gene-centric analysis an additional file defining gene names and coordinates is required. This file is a white-space delimited file (space or tab) and illustrated in Table 3. The software download includes the gene files for both build 35 and 36 of the genome.

**Table 1:** Three Column Map File

*CHR*	*MARKER*	*BP POS*
1	SNP1	1111
1	SNP2	2111
1	SNP3	3111
1	SNP4	4111

3 column map file that contains the chromosome, marker name, and base pair position of each marker. The header row is for display purposes only and should not appear in the actual file.

**Table 2:** Four Column Map File

*CHR*	*MARKER*	*GEN POS*	*BP POS*
1	SNP1	0	1111
1	SNP2	1	2111
1	SNP3	2	3111
1	SNP4	3	4111

4 column map file that contains the chromosome, marker name, genetic position, and base pair position. The four column format is the same used by the genetics software package PLINK. The header row is for display purposes only and should not appear in the actual file.

**Table 3:** Pedigree File

*PED ID*	*INDIV ID*	*FATHER ID*	*MOTHER ID*	*SEX*	*AFF STAT*	*GENOTYPES*					
1	1	0	0	1	1	A	A	A	C	T	G
2	2	0	0	2	1	A	G	A	A	G	G
3	3	0	0	2	2	G	G	C	C	T	T
4	4	0	0	1	2	A	G	A	C	T	G

Pedigree file that contains genotype data for 3 SNPs and 4 individuals (2 controls and 2 cases). The first column is for pedigree IDs, the second for individual IDs, the third for paternal ID, the forth for maternal ID, the fifth for sex code, and the sixth for disease designation or quantitative phenotype value. Column 7 starts the genotype data for the markers, with each allele of each genotype in its own column (e.g. for 3 markers there will be 6 allele columns). The header row is for display purposes only and should not appear in the actual file.

### Program Options

CCRaVAT and QuTie provide users with 25 command-line options, all detailed in the users manual, allowing the analysis to be tailored to specific interests. The options belong to three broad categories: altering the definitions of a region, low frequency/rare variant; altering significance levels and defining statistical analysis method, and altering the appearance of the graphical output.

Fundamental to the collapsing method is the definition of the region within which the accumulation of low frequency/rare variants will be examined. CCRaVAT and QuTie provide the user with two options for defining the locus of interest, either through defining regions based on known gene coordinates or by employing a sliding window approach. If the analysis is based on sliding windows, the user defines how large the analysis windows should be. If a gene-based analysis is undertaken the user can also define how further upstream and downstream from the transcription start and stop sites to extend the analysis. The user can adjust the MAF cut-off that determines which markers are considered to be low frequency/rare variants and therefore included in the analysis.

Unlike association tests of common variants, there is no well-defined significance threshold for the analysis of multiple low frequency/rare variants. The programs allow the user to define a significance threshold that produces separate files for significant regions, allowing the researcher to focus on top hits without having to troll through all the data. The researcher can also set significance thresholds to select regions for follow-up by undergoing permutation analysis. The number of permutations can also be preset. As chi-squared test results can be unreliable with low cell counts, CCRaVAT provides an option for the user to set a minimum number of cell counts; the Fisher's exact test is then implemented for any region that falls below this value. The standard analysis of QuTie is a linear regression, but QuTie provides an option to additionally carry out a two-sample t-test.

To assist researchers in interpreting the results, CCRaVAT and QuTie produce visual output summaries. The programs allow the user to define a significance threshold to highlight loci in the Manhattan plot on the basis of their p value, as well as to manipulate graphical parameters such as the height, width, and size of data points of the figures. The programs also provide an option to (re)produce figures based on previously run analyses.

### Output Files

**Figure 2:**
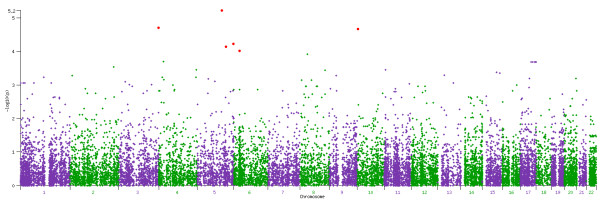
**CCRaVAT and QuTie Manhattan Plot**. An example Manhattan plot generated by CCRaVAT and QuTie displaying the -LOG10 p value of all genes/windows analyzed. Each point represents a gene or region, with loci achieving p values below a predefined threshold denoted in red.

**Figure 3:**
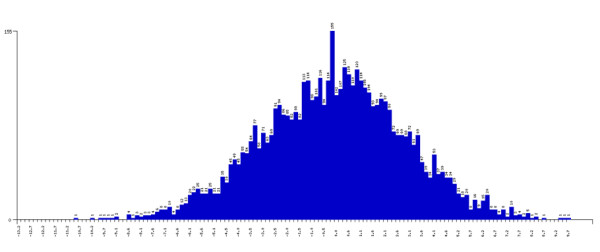
**QuTie Quantitative Trait Distribution Histogram**. Histogram showing the distribution of the analysed quantitative trait across all individuals (individuals with and without low frequency/rare-variant minor alleles).

**Figure 4:**
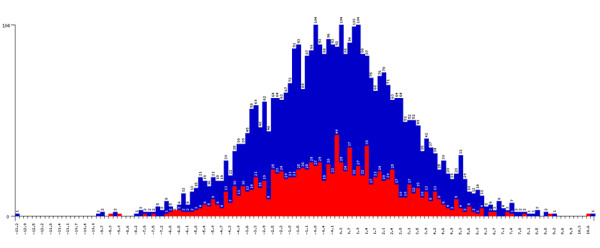
**QuTie Quantitative Trait Distribution Comparison Histogram**. Histogram displaying the distribution of quantitative trait values for individuals that either do (red) or do not (blue) carry at least one low frequency/rare variant minor allele within a region that has a p value ≤ the value set by the -pout option. A histogram is produced for every significant gene/window.

CCRaVAT and QuTie produce text-based summaries and graphical summaries of the analysis results. The format of the CCRaVAT output file that provides summary statistics for all genes/windows that achieved a user-specified level of significance is displayed in Table 5. The same summary file produced by QuTie is illustrated in Table 6. The results of permutation testing for all regions that reached the significance threshold are demonstrated in Table 7. CCRaVAT and QuTie produce comprehensive output including summary statistics for all analysed genes/windows on each chromosome and this output is summarized in Tables 8 and 9 (respectively). The programs also produce a list of SNPs that were analyzed within each significant region, and the format of that file is shown in Table 10. In addition to these output files, CCRaVAT and QuTie produce a Manhattan plot that visually summarizes the significance of all analyzed regions (Figure 2). QuTie produces two additional graphic summaries (Figures 3 and 4). The histogram shown in Figure 3 shows the distribution of quantitative trait values for all individuals in the pedigree file. Figure 4 is an example of the histogram that QuTie produces for every region achieving a user-specified level of significance, and shows the distribution of trait values of individuals with (red) and without (blue) low frequency/rare variant minor alleles. The output for a genome-wide, gene-centric scan for low frequency/rare variant (MAF≤0.05) analysis typically totals less than 2 Mb for all files. The output size for sliding windows-based analysis genome-wide depends on the size of the intervals examined and the MAF threshold imposed. This usually ranges from 3 to 6 Mb for all files.

**Table 4:** Gene File

*GENE ID*	*GENE NAME*	*CHR*	*START BP POS*	*STOP BP POS*
7293	TNFRSF4	1	1136569	1139375
51150	SDF4	1	1142151	1157274
126792	B3GALT6	1	1157508	1160281
388581	C1QDC2	1	1167696	1171965
118424	UBE2J2	1	1179155	1199097
6339	SCNN1D	1	1207439	1217272
116983	CENTB5	1	1218807	1228503
126789	PUSL1	1	1233857	1236920

Gene file that defines the genes to be analyzed and their coordinates to allow the collapsing of the correct markers defined in the map file. The first five columns of the file must be: Gene ID, Gene Name/Symbol, Chromosome, Start bp position, End bp position. Additional columns will be ignored.

**Table 5:** CCRaVAT Summary Output File

*Gene/Wind*	*Chr*	*Start*	*End_Pos*	*N_SNPs*	*CaseRV*	*CaseNoRV*	*ContRV*	*ContNoRV*	*ChiSq*	*P-val*	*FisherExPval*
MGC33212	3	197456409	197583455	(10/1)	2	1909	31	2903	15.5	0.000083	1.96E-05
PPIC	5	122336979	122450324	(12/6)	26	1877	7	2906	21.44	0.0000037	5.94E-06
NR3C1	5	142589325	142813087	(24/2)	8	1912	47	2869	14.71	0.00013	7.28E-05
ADAMTS2	5	178423474	178754935	(30/2)	62	1859	44	2885	16.15	0.000059	No < 30
3.8_1.5	6	29790873	29892049	(43/2)	26	1890	10	2920	16.21	0.000057	9.74E-05
KLF6	10	3761233	3867455	(14/2)	14	1907	1	2931	18.18	0.00002	2.13E-05

This file provides summary statistics for all genes that achieved a p value ≤ the p value set by the -pout command line option. The summary file is a tab-delimited file with 12 columns: Gene/Window name, Chromosome, Starting bp position, End bp position, Number of SNPs in the Gene/Window, Number of cases with low frequency/rare variant minor alleles, Number of cases without low frequency/rare variant minor alleles, Number of controls with low frequency/rare variant minor alleles, Number of controls without low frequency/rare variant minor alleles, Chi-Squared Value, Chi-Squared p value, Fisher exact p value.

**Table 6:** QuTie Summary Output File

*Gene*	*Chr*	*Start*	*End_pos*	*SNPs/rvSNPs*	*QT+RV*	*QT-RV*	*QT+RV mean*	*QT-RV mean*	*p-val*	*BetaCoef*	*St.Er*	*[lowCI - upCI]*	*t-test*	*t-test_p-val*
MIB2	1	0	107622	(10/1)	106	1125	-0.367	0.037	6.77E-05	0.404	0.101	[0.206 - 0.602]	-3.975	3.72E-05
GOLGA8C	15	18977714	19091040	(29/4)	127	1097	-0.347	0.042	3.19E-05	0.389	0.093	[0.206 - 0.571]	-4.149	1.78E-05
TPO	2	1346242	1575502	(29/6)	70	1158	0.478	-0.028	4.00E-05	-0.506	0.122	[-0.746 - -0.266]	4.107	1.80E-05
EXOC3	5	446375	570407	(24/1)	4	1228	1.935	-0.006	1.00E-04	-1.941	0.498	[-2.918 - -0.964]	3.874	5.12E-05
C10orf110	10	1008606	1130138	(66/4)	111	1116	0.379	-0.036	4.00E-05	-0.415	0.099	[-0.609 - -0.221]	4.161	1.48E-05

This file provides summary statistics for all genes that achieved a p value ≤ the p value set by the -pout command line option. The summary file is a tab-delimited file with 15 columns: Gene/Window name, Chromosome, Starting bp position, End bp position, Number of SNPs in the Gene/Window, Number of individuals with low frequency/rare variant minor alleles, Number of individuals without low frequency/rare variant minor alleles, The mean QT value of individuals with low frequency/rare variant minor alleles, The mean QT value of individuals without low frequency/rare variant minor alleles, The p value of the linear regression, The Beta coefficient from the linear regression, The Standard Error of the Beta Coefficient, the lower and upper 95% confidence intervals of Beta, The t-statistic, and The t-statistic p value.

**Table 7:** CCRaVAT Permutation Summary Output File

*Gene/Window*	*Chr*	*Start*	*End*	*Case: (RV/NonRV)* *Control: (RV/NonRV)*	*Pval*	*Permutation Results*
LOC254099	1	1012320	1219359	case: (86/1213) cont: (75/581)	0.00026	Perm: 0/10 = 0
TTLL10	1	1055000	1261164	case: (164/1133) cont: (104/549)	0.047	Perm: 0/10 = 0
TNFRSF18	1	1078812	1282012	case: (164/1133) cont: (104/549)	0.047	Perm: 1/10 = 0.1
TNFRSF4	1	1086630	1289435	case: (164/1133) cont: (104/549)	0.047	Perm: 1/10 = 0.1
SDF4	1	1092212	1307334	case: (164/1133) cont: (104/549)	0.047	Perm: 0/10 = 0
B3GALT6	1	1107568	1310341	case: (164/1133) cont: (104/549)	0.047	Perm: 1/10 = 0.1
C1QDC2	1	1117751	1318766	case: (164/1133) cont: (104/549)	0.047	Perm: 0/10 = 0
UBE2J2	1	1129217	1349157	case: (164/1133) cont: (104/549)	0.047	Perm: 0/10 = 0
SCNN1D	1	1157499	1367332	case: (164/1133) cont: (104/549)	0.047	Perm: 0/10 = 0

This file provides summary statistics for all genes that achieved a p value ≤ the p value set by the -pperm command line option, which initiates permutation testing. The summary file is a tab-delimited file with 8 columns: Gene/Window name, Chromosome, Starting bp position, End bp position, Summary of the number of cases and controls that have low frequency/rare variant minor alleles, the original p value, Summary of permutations run, and Permutation p value. The output file for QuTie is the same except that column 5 contains the number of individuals with and without low frequency/rare variant minor alleles and corresponding QT values.

**Table 8:** CCRaVAT Chromosome Output File

*Gene*	*Chr*	*Start*	*Stop*	*N_SNPs*	*Case+RV*	*Case-RV*	*Cont+RV*	*Cont-RV*	*Chisq*	*PearsonPval*	*FishExPval*	*Permutations*
MIB2	1	0	107622	(5/0)	0	1924	0	2938	0	1	1	
OR4G11P	1	2878	103747	(6/1)	1	1922	2	2935	0.05	0.82	1	
MMP23B	1	9202	111672	(0/0)	0	1924	0	2938	0	1	1	
MMP23A	1	9225	111672	(12/2)	112	1784	167	2755	0.08	0.78	No < 30	
CDC2L2	1	12742	197336	(32/1)	1	1921	7	2926	2.46	0.12	0.158	
LOC440748	1	39316	143660	(47/12)	380	1531	594	2329	0.14	0.71	No < 30	
NBPF20	1	114476	233524	(23/1)	4	1919	2	2932	1.84	0.17	0.222	
CCNL2	1	115136	226993	(13/0)	0	1924	0	2938	0	1	1	
OR4F29	1	357522	544452	(7/3)	159	1756	239	2685	0.03	0.86	No < 30	
LOC440551	1	519055	657573	(5/0)	0	1924	0	2938	0	1	1	
LOC440552	1	558787	660167	(29/9)	207	1679	263	2640	4.74	0.029	No < 30	Perm: 1/10 = 0.1
FAM87B	1	742614	845077	(28/3)	15	1906	21	2907	0.06	0.81	0.865	

This file provides summary statistics for all genes analyzed on each chromosome and is the most comprehensive output file. The summary file is a tab-delimited file with 13 columns: Gene/Window name, Chromosome, Starting bp position, End bp position, Number of SNPs in the Gene/Window and the number that are low frequency/rare, Number of cases with low frequency/rare variant minor alleles, Number of cases without low frequency/rare variant minor alleles, Number of controls with low frequency/rare variant minor alleles, Number of controls without low frequency/rare variant minor alleles, Chi-Squared Value, Chi-Squared p value, Fisher exact p value, and a description of any permutations run.

**Table 9:** QuTie Chromosome Output File

*Gene*	*Start*	*End*	*(SNPs/RV)*	*QT+RV*	*QT-RV*	*QT+RV_Av*	*QT-RV_Av*	*RegPval*	*BetaCoef*	*StEr*	*[lCI - uCI]*	*T*	*Ttest_Pval*
MIB2	924412	1025537	(20/1)	4	597	9.014	-0.137	0.222	-9.151	7.484	[-23.819 - 5.517]	1.223	0.111
OR4G11P	938946	1039986	(25/1)	4	597	9.014	-0.137	0.222	-9.151	7.484	[-23.819 - 5.517]	1.223	0.111
MMP23B	945587	1081419	(43/6)	75	281	3.308	-0.354	0.06	-3.662	1.94	[-7.484 - 0.159]	1.884	0.03
MMP23A	983671	1097098	(46/7)	89	280	2.929	-0.493	0.059	-3.423	1.807	[-6.983 - 0.138]	1.89	0.03
CDC2L2	997120	1097869	(43/7)	89	280	2.929	-0.493	0.059	-3.423	1.807	[-6.983 - 0.138]	1.89	0.03
LOC440748	1007128	1117407	(47/8)	115	260	2.73	-0.135	0.085	-2.865	1.659	[-6.134 - 0.404]	1.724	0.04
NBPF20	1062320	1169359	(32/10)	159	224	0.713	-0.169	0.558	-0.882	1.505	[-3.847 - 2.082]	0.588	0.28
CCNL2	1105000	1211164	(35/14)	150	523	-1.291	0.224	0.281	1.515	1.403	[-1.235 - 4.266]	-1.081	0.14
OR4F29	1128812	1232012	(30/17)	142	437	-1.402	0.231	0.258	1.633	1.442	[-1.193 - 4.459]	-1.133	0.13
LOC440551	1136630	1239435	(30/17)	143	443	-1.407	0.204	0.262	1.611	1.435	[-1.201 - 4.423]	-1.123	0.13
LOC440552	1142212	1257334	(28/15)	139	444	-1.48	0.156	0.261	1.636	1.454	[-1.214 - 4.485]	-1.126	0.13
FAM87B	1157568	1260341	(22/14)	110	449	-1.612	0.209	0.254	1.822	1.596	[-1.306 - 4.949]	-1.142	0.13

This file provides summary statistics for all genes analyzed on each chromosome and is the most comprehensive output file. The summary file is a tab-delimited file with 14 columns: Gene/Window name, Starting bp position, End bp position, Number of SNPs in the Gene/Window, Number of individuals with low frequency/rare variant minor alleles, Number of individuals without low frequency/rare variant minor alleles, Mean QT value for individuals with low frequency/rare variant minor alleles, Mean QT value for individuals without low frequency/rare variant minor alleles, Linear regression p value, Beta coefficient from linear regression, Standard Error of the Beta coefficient, The lower and upper 95% Confidence Interval s of Beta, T-statistic, and T-statistic p value.

**Table 10:** CCRaVAT/QuTie Significant Region Output File

*Marker*	*Chromosome*	*Position*	*MAF*
rs715643	1	1212830	0.042
rs3934834	1	1045729	0.163
rs3737728	1	1061338	0.301
rs6687776	1	1070488	0.158
rs9651273	1	1071463	0.295
rs4970405	1	1088878	0.086
rs12726255	1	1089873	0.125
rs2298217	1	1104902	0.133
rs4970357	1	1116987	0.093
rs4970362	1	1134661	0.378
rs9660710	1	1139265	0.068
rs4970420	1	1146396	0.192
rs1320565	1	1159781	0.095
rs11260549	1	1161717	0.116
rs9729550	1	1175165	0.262
rs11721	1	1192554	0.101
rs2887286	1	1196054	0.17
rs3813199	1	1198200	0.106
rs3766186	1	1202358	0.105
rs7515488	1	1203727	0.158
rs6675798	1	1216520	0.105

This file provides summary statistics for all SNPs that reside within a gene or region with p value ≤ the p value set by the -pout command line option. The file is tab-delimited with 5 columns: Marker name, Chromosome, bp position of Marker, and the Minor Allele Frequency (MAF) considering all analyzed individuals.

### Data Quality Control

Performing the collapsing analysis based on low frequency and rare variants (particularly those typed as part of GWAS) requires special attention to quality control. Genotype calling algorithms for GWAS chips perform well for common variants, but are known to be error-prone for loci with low MAF. Therefore, we recommend users that have performed the analysis based on GWAS chip data to check the cluster plots for all variants contributing to interesting signals, exclude any poorly clustering variants and rerunning the analysis for the specific regions of interest to ensure the association is robust to these exclusions. Quality control is also an important consideration when analyzing sequencing data. Major considerations are the effects of small insertions-deletions leading to false positive SNPs, read depth at variant sites, mapping quality score, and SNP quality score.

## Conclusions

In this paper we have described two novel analysis tools, CCRaVAT and QuTie, for investigating low frequency/rare variant associations in GWAS and resequencing data. Both programs employ a simple collapsing method to increase power over single point analysis. CCRaVAT analyzes case/control data and investigates significance using Pearson's chi-squared and Fisher's exact tests. QuTie analyzes quantitative trait data and implements a linear regression and Student's t-test. Both CCRaVAT and QuTie are easy-to-use Linux command line tools that use standard files typically employed in common variant GWAS analysis. CCRaVAT and QuTie can be used as a complement to existing common disease GWAS by analyzing low frequency/rare variant associations or in analyzing sequence-based low frequency/rare variant genotype calls in regions of interest or genome-wide. These tools are important first steps in the analysis of rare variants. We are currently developing more powerful natural extensions to the current methods as well as novel approaches that incorporate weights based on quality metrics.

## Availability and requirements

**Project name**: CCRaVAT and QuTie

**Project homepage**: http://www.sanger.ac.uk/resources/software/rarevariant/

**Operating system**: Linux/Unix

**Programming Language**: Perl

**License**: GNU GPL

## List of abbreviations

CCRaVAT: case control rare variant analysis tool; QuTie: quantitative trait; QT: quantitative trait; GWAS: genome-wide association study; SNP: single nucleotide polymorphism; MAF: minor allele frequency; bp: base-pair; CHR: chromosome; POS: position; GEN: genetic; AFF STAT: affection status; RV: rare variant; Cont: control; ChiSq: Chi-square statistic; FisherEX: Fisher's exact test; Wind: window; Coef: coefficient; StEr: standard error; CI: confidence interval; Av: average; RegPval: regression p-value.

## Authors' contributions

RL wrote the code for CCRaVAT and QuTie. ADW wrote the documentation, developed the homepage, and drafted the manuscript. KSE compiled and created the gene files for the gene-centric analysis. APM supervised the development of CCRaVAT and QuTie. EZ supervised the development of CCRaVAT and QuTie and drafted the manuscript. All authors have read and approved this manuscript.
